# Excellent Response to Eculizumab in a Patient With Complement‐Mediated Thrombotic Microangiopathy and a Novel Plasminogen Gene Variant

**DOI:** 10.1155/crh/1318626

**Published:** 2026-10-12

**Authors:** Aman Patel, Bowen Powers, Jason Tsai, Suresh Nukala

**Affiliations:** ^1^ Mercer University School of Medicine Columbus Georgia USA mercer.edu; ^2^ Nephrology Associates of Columbus Columbus Georgia USA; ^3^ Piedmont Columbus Midtown Hospital Columbus Georgia USA

## Abstract

A middle‐aged man presented with myalgia, fever, hematemesis, and melena. He was found to have acute kidney injury, acute coronary syndrome, thrombocytopenia, and Coombs‐negative hemolytic anemia, raising concern for thrombotic microangiopathy (TMA). While awaiting confirmatory testing, empirical plasma exchange was initiated. Initially, minimal red blood cell fragmentation was observed, which later progressed to florid fragmentation. Despite supportive care, his clinical status deteriorated requiring hemodialysis. The presence of TMA, along with *ADAMTS13* metalloprotease activity above the diagnostic threshold, worsening kidney function, and negative stool Shiga toxin testing suggested complement‐mediated TMA (C‐TMA). Stool PCR testing revealed rotavirus. Eculizumab was initiated, resulting in rapid normalization of hematological parameters and eventual renal recovery. Genetic testing identified a heterozygous *PLG* c.1735G > A (p.Gly579Arg) variant classified as a variant of uncertain significance (VUS). The importance of serial review of peripheral blood smears and the diagnostic challenge of differentiating TTP, STEC‐HUS, and C‐TMA are the key learning points.

## 1. Background

Thrombotic microangiopathy (TMA) is a clinical syndrome characterized by microangiopathic hemolytic anemia (MAHA), thrombocytopenia, and ischemic organ injury due to endothelial damage and subsequent microthrombus formation in arterioles [1]. MAHA, the hallmark of TMA, is a Coombs‐negative hemolytic anemia with red blood cell (RBC) fragmentation. Platelets are consumed in microthrombus formation, and excess shear stress results in fragmentation of RBCs, producing hemolytic anemia.

TMA can be broadly classified as primary or secondary. Primary TMA includes thrombotic thrombocytopenic purpura (TTP), Shiga toxin–producing *Escherichia coli* hemolytic uremic syndrome (STEC‐HUS), and complement‐mediated TMA (C‐TMA). Patients with secondary TMA have identifiable underlying conditions including malignancy, autoimmune disorders, exposure to chemotherapeutic agents or calcineurin inhibitors, or a background of solid organ or hematopoietic stem cell transplantation [2, 3].

TTP is caused by a severe deficiency of *ADAMTS13*, a zinc‐containing metalloprotease (a disintegrin and metalloprotease with a thrombospondin Type 1 motif, Member 13) that cleaves ultralarge von Willebrand factor (UL‐VWF) multimers. Inability to cleave these multimers results in accumulation of UL‐VWF on the endothelium, leading to thrombus formation, platelet consumption, and MAHA. An *ADAMTS13* activity of less than 10% is diagnostic of TTP. Approximately 95% of TTP cases are caused by autoantibody formation against *ADAMTS13* (immune TTP), for which plasma exchange is the cornerstone of acute management. The remaining 5% are caused by biallelic mutations of the *ADAMTS13* gene (congenital TTP), which is managed with recombinant ADAMTS13 or plasma infusion rather than plasma exchange. If left untreated, TTP carries a mortality of up to 90% [2].

STEC‐HUS is a form of TMA caused by infections with Shiga toxin–producing organisms, most commonly *E. coli* O157:H7 but also *Salmonella* and *Shigella* [4]. Shiga toxin binds to globotriaosylceramide (Gb3) receptors prominently expressed on glomerular endothelial cells, causing direct endothelial injury, platelet aggregation, and renal failure. Children aged under 5 years are most frequently affected [5], though adult cases occur. Transmission is typically through ingestion of contaminated meat or dairy products and rarely by human‐to‐human spread. A diarrheal prodrome precedes STEC‐HUS by 5–10 days in approximately 95% of patients [6], and multisystem involvement is less common than in C‐TMA [7]. Transient reductions in C3 and elevations in soluble C5b‐9 can occur due to secondary complement activation, though these findings are nonspecific and nondiagnostic for either STEC‐HUS or C‐TMA [8]. Shiga toxin is detectable in approximately 75% of patients if stool cultures and PCR are performed promptly; however, detection is transient at approximately 7 days in adults [9]. Shiga toxin–negative infection‐associated HUS—caused by organisms such as *Streptococcus pneumoniae*—accounts for approximately 10% of cases [10]. *ADAMTS13* activity exceeds 10% in STEC‐HUS. Majority of patients respond to supportive care [11], and lack of response should raise concern for C‐TMA, prompting initiation of C5 inhibitor therapy to avoid irreversible kidney injury [12]. The incidence of STEC‐HUS varies by geography; in France, it is estimated at 10 per million [13]. Relapses are uncommon in STEC‐HUS.

C‐TMA (previously termed atypical HUS) is the diagnosis considered in patients with TMA in whom *ADAMTS13* activity exceeds 10%, Shiga toxin testing is negative, and no underlying secondary cause is identified [3]. Complement dysregulation is the central mechanism. Approximately 50% of patients have an identifiable hereditary pathogenic mutation in a complement regulatory gene, involving either gain‐of‐function mutations increasing C3 or complement factor B (CFB) activity, or loss‐of‐function mutations reducing the activity of complement factor H (CFH), complement factor I (CFI), or membrane cofactor protein (MCP). A further 10% of cases are acquired, related to autoantibodies against CFH [14–17]. The remaining 40% of patients with C‐TMA have no identifiable pathogenic mutation or carry a variant of uncertain significance (VUS). The epidemiology of C‐TMA remains incompletely characterized, with an estimated adult prevalence of 2–10 per million [18, 19]. Although historically considered a pediatric disease, it is now recognized that 58% of cases occur in adults and 42% in children, typically adolescents [20, 21]. Relapse following discontinuation of C5 inhibitor therapy occurs in approximately 40%–60% of patients with identified pathogenic mutations and in less than 5% of those without an identifiable pathogenic mutation or autoantibody [22, 23].

## 2. Case Presentation

A 49‐year‐old man presented with a 3‐day history of severe generalized myalgias and fever up to 39.4°C. On the day of admission, he had coffee ground emesis and melanotic stool. He had new‐onset numbness in his toes, and he reported a nonspecific sense of derealization and perceptual alteration. One month prior to presentation, he had visited the Caribbean. Soon after returning, he had a transient illness comprising weakness, bloody emesis, and melena which lasted 4 days.

His medical history was unremarkable. He had a 22 pack‐year smoking history (quit six years prior), no excessive alcohol or recreational drug use, and no family history of hematological conditions, renal failure, or TMA. Both parents had skin cancer.

He was tachycardic (heart rate: 105 bpm), hypotensive (blood pressure: 97/62 mmHg), mildly confused, and appeared ill. Physical examination showed petechiae on the right upper extremity and oropharyngeal thrush, with no lymphadenopathy, hepatosplenomegaly, or abdominal tenderness.

## 3. Investigations

Admission laboratory tests indicated acute kidney injury, transaminitis, Type 2 myocardial infarction, and severe thrombocytopenia, suggestive of multiple‐organ dysfunction. Electrocardiogram showed sinus tachycardia and nonspecific T‐wave abnormalities. Noncontrast computed tomography of the chest, abdomen, and pelvis was unremarkable.

Unexplained multiorgan failure, severe thrombocytopenia, and anemia led to TMA being considered. Infectious workup, prompted by recent travel, showed rotavirus‐positive stool on PCR. Tests for *E. coli* O157:H7, Shiga toxin, HIV, leptospirosis, hepatitis A and B, malaria, dengue virus, Babesia, COVID‐19, *Rickettsia*, and *Anaplasma* were negative.

TMA workup included *ADAMTS13* activity, lactate dehydrogenase (LDH), haptoglobin, and complement levels. Peripheral blood smear showed rare RBC fragments (1 per high‐power field) (Figure 1 Panel A). LDH was markedly elevated at 2727 U/L (normal: 140–280 U/L), platelet count was 9 × 10^9^/L, hemoglobin (Hb) was 9.9 g/dL, hematocrit was 0.27, and haptoglobin was undetectable (< 30 mg/dL; normal: 44–215 mg/dL). The direct Coombs test was negative. Prothrombin time was minimally prolonged (15.9 s; normal: 11–13.5 s), INR was 1.28 (normal: < 1.20), and partial thromboplastin time was 39.6 s (normal: 25–35 s). D‐dimer was markedly elevated (> 20 μg/mL; normal: < 0.5 μg/mL), and fibrinogen was elevated at 546 mg/dL (normal: 200–400 mg/dL). Aspartate aminotransferase (AST) and alanine aminotransferase (ALT) were elevated at admission and subsequently normalized with treatment (specific values are given in Table 1).

**Figure 1:**
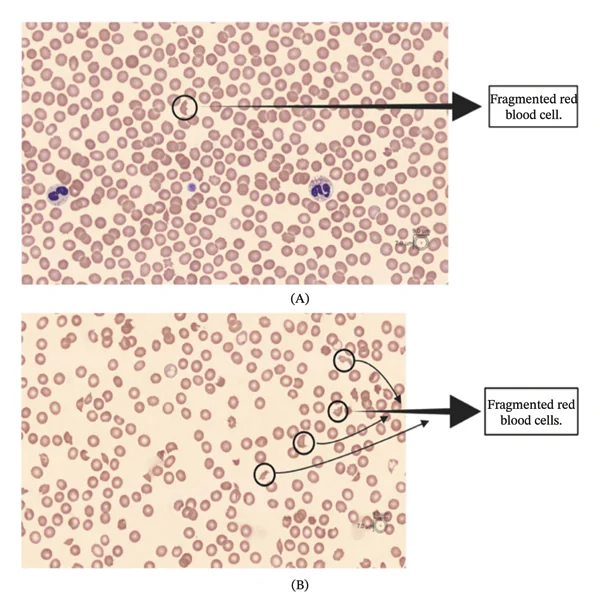
Peripheral blood smear of the patient on Day 1 showing 1–2 red blood cell fragments per high‐power field (Panel A) and Day 3 peripheral blood smear showing > 15 red blood cell fragments per high‐power field (Panel B).

**TABLE 1:** Laboratory trends over time.

Lab (normal range)	Day 0: ED	Day 1	Day 2: plasmapheresis initiated	Day 3 dialysis initiated	Day 5	Day 6: eculizumab initiated	Day 9	Follow‐up: 5 weeks
Hemoglobin 13.8–17.2 g/dL	14.7	12.2	9.9	7.6	7.5	8.1	8.2	12.9
Mean corpuscular volume 81–98 fL	86							
White blood cells 4.5–11.0 × 10^9^/L	21.7	17.1	14.7	10.7	33.5	39	18.4	6.9
Platelets 150–450 × 10^9^/L	19	15	9	15	40	51	150	326
Blood urea nitrogen: 6–24 mg/dL	73	88	134	163	78	50	110	12
Creatinine: 0.74–1.35 mg/dL	6.8	7.0	9.9	10.1	5.9	4.1	7.7	1.1
Alanine aminotransferase 7–55 U/L	150	116	67	27	17	13	12	14
Aspartate aminotransferase 8–48 U/L	221	192	96	46	20	17	13	14
Prothrombin time: 11–13.5 s	15.9	14.8						
International normalized ratio: 0.8–1.2	1.28	1.17						
Partial thromboplastin time: 25–35 s	39.6	34.6						
Bilirubin: 0.1–1.2 mg/dL	1.3	2.2	1.9		1.2	1.0	0.5	0.2
Lactate dehydrogenase: 120–280 U/L		2727			637		247	183
Urinalysis	3+ blood, 3+ protein, 13 RBC, 17 WBC							
Troponin: < 14 ng/L	5073							
Complement C3: 88–206 mg/dL			80					
Complement C4: 19–52 mg/dL			30					
ADAMTS13: 0.68–1.63 IU/mL						0.67		
sC5b‐9: ≤ 250 ng/mL							462	
Microbiology			Rotavirus RNA detected					

Complement testing showed low C3 (80 mg/dL; normal: 88–206 mg/dL) and normal C4 (30 mg/dL; normal: 19–52 mg/dL). CH50 was normal (45 U/mL; 31–60 U/mL), soluble C5b‐9 was elevated (462 ng/mL; < 250 ng/mL), Factor H level was normal, and Factor H autoantibody assay (FHAA) was negative. Antinuclear antibody (ANA), rheumatoid factor, anticardiolipin, beta‐2‐glycoprotein I antibodies, and lupus anticoagulant were negative.

## 4. Differential Diagnosis

1. TTP. TTP was the primary diagnostic concern at presentation given the combination of MAHA, severe thrombocytopenia, neurological symptoms (derealization, toe numbness), and a PLASMIC score of 6, indicating high pretest probability. Florid RBC fragmentation on the follow‐up smear further supported this possibility. However, several features were atypical: Severe anuric renal failure is uncommon in TTP, where kidney involvement is generally mild (creatinine typically below 2.5 mg/dL), and the concurrent Type 2 myocardial infarction suggested a more systemic microvascular process. Ultimately, an *ADAMTS13* activity of 0.67 IU/mL—above the diagnostic threshold of 0.10 IU/mL—excluded TTP as the primary diagnosis.
2. STEC‐HUS. STEC‐HUS was considered given the patient’s recent travel to the Caribbean, gastrointestinal symptoms, and multiorgan involvement. However, several features argued against this diagnosis. Stool testing for Shiga toxin and *E. coli* O157:H7 was negative. While a negative Shiga toxin test does not exclude STEC‐HUS—the toxin is detectable in only approximately 75% of cases, and detection is transient at around seven days in adults—the absence of a prolonged diarrheal prodrome (which typically precedes STEC‐HUS by 5–10 days in 95% of patients) and the patient’s adult age reduced this probability further.
3. C‐TMA. Several features supported consideration of C‐TMA: severe acute kidney injury requiring dialysis, multiorgan involvement (renal, cardiac, and neurological), low C3 with normal C4, and elevated soluble C5b‐9. However, soluble C5b‐9 is nonspecific and cannot establish or exclude C‐TMA in isolation. Two features were atypical. First, hypertension, common in C‐TMA, was absent; the patient was instead hypotensive, likely reflecting his concurrent Type 2 myocardial infarction, gastrointestinal bleeding, and systemic inflammatory response. Second, the platelet nadir of 9 × 10^9^/L was lower than typically observed in C‐TMA and may have been influenced by concurrent coagulopathy, given the mildly prolonged prothrombin time and markedly elevated D‐dimer. These atypical features illustrate the clinical heterogeneity of TMA and should not, in isolation, exclude C‐TMA
4. Secondary TMA—other causes. Several causes of secondary TMA were considered and excluded. Catastrophic antiphospholipid syndrome (CAPS) can produce acute multiorgan thrombosis with low complement levels and MAHA, often triggered by infection [24], but was excluded by negative lupus anticoagulant, anticardiolipin, and beta‐2 glycoprotein I antibodies. Systemic lupus erythematosus (SLE) may cause thrombocytopenia, low complement, and renal failure but was considered unlikely given the negative ANA and absence of characteristic features including arthralgia, alopecia, and facial rash. HIV‐associated TMA and SARS‐CoV‐2–associated multiorgan dysfunction were excluded by negative serological testing. Disseminated intravascular coagulation (DIC) was considered early given the coagulopathy, but the mildly prolonged prothrombin time combined with an elevated fibrinogen—rather than the hypofibrinogenemia characteristic of acute DIC—made this diagnosis unlikely [25]. Although these findings did not completely exclude a concurrent coagulopathy, they provided limited support for a mixed TMA/coagulopathy phenotype.
5. An infection‐associated TMA was also considered in the setting of rotavirus infection and systemic illness. However, the patient’s progressive renal‐predominant TMA, severe multiorgan involvement, and continued deterioration despite supportive care and three sessions of plasma exchange were less typical of TMA that would be expected to improve with resolution of the underlying infection. Although rotavirus may have contributed to the acute illness, the available data do not establish whether it triggered or directly contributed to the TMA. Overall, the combination of severe renal involvement requiring dialysis, multiorgan disease, complement abnormalities, and exclusion of alternative primary and secondary causes favored C‐TMA as the leading diagnosis.

## 5. Treatment

Given the clinical presentation—severe thrombocytopenia, MAHA, and neurological symptoms—TTP was the primary working diagnosis. The PLASMIC score, a validated seven‐point clinical prediction tool estimating the probability of severe *ADAMTS13* deficiency in patients presenting with TMA, was calculated at six out of seven, indicating high risk for TTP [26]. This patient’s score was based on the following components: platelet count less than 30 × 10^9^/L (1 point), evidence of hemolysis with undetectable haptoglobin (1 point), no active cancer (1 point), no prior solid organ or stem cell transplant (1 point), MCV less than 90 fL (1 point), INR less than 1.5 (1 point), and creatinine above 2.0 mg/dL precluding the seventh point. The resulting score of six out of seven falls in the high‐risk category (72% probability of severe ADAMTS13 deficiency).

Consistent with current guidelines, urgent plasma exchange was initiated on Day 2 following vascular catheter placement and informed consent, without awaiting ADAMTS13 results, given the high pretest probability and well‐established mortality risk of untreated TTP. The risks of plasma exchange—including bleeding, thrombosis, hypotension, and arrhythmia—were carefully considered in the context of his concurrent acute coronary syndrome [27]. Given the high pretest probability of TTP and the pending ADAMTS13 result, intravenous methylprednisolone (1000 mg daily for three days) was initiated as adjunctive therapy for presumed immune TTP, with sucralfate and pantoprazole added for gastroprotection in the context of his recent upper gastrointestinal bleeding. He was subsequently transitioned to oral prednisone (30 mg twice daily).

On Day 3, the patient’s clinical status deteriorated significantly. He developed worsening hypoxia requiring supplemental oxygen at 50% FiO_2_, hypotension necessitating norepinephrine infusion, and persistent anuria with progressive metabolic acidosis, prompting initiation of hemodialysis. He received packed RBC transfusions when Hb fell below 7 g/dL. A follow‐up peripheral blood smear showed florid RBC fragmentation (Figure 1 Panel B), representing progression from the rare fragments seen on the initial smear. After three sessions of plasma exchange, his platelet count had risen to 40 × 10^9^/L, liver enzymes had normalized, and LDH had declined to 637 U/L with creatinine at 5.06 mg/dL—a partial response that remained insufficient given his ongoing renal failure and clinical instability.

On Day 6, the *ADAMTS13* activity returned at 0.67 IU/mL (normal: 0.68–1.63 IU/mL), which, while just below the normal reference range, was well above the diagnostic threshold of 0.10 IU/mL, making TTP unlikely. With negative Shiga toxin testing and progressive renal failure despite plasma exchange, C‐TMA became the leading diagnostic consideration. Soluble C5b‐9 and the Invitae Atypical Hemolytic Uremic Syndrome and Thrombotic Microangiopathies Panel (CFH, CFHR1, CFHR2, CFHR3, CFHR4, CFHR5, CFI, CFB, C3, CD46/MCP, THBD, PLG, and DGKE) were obtained. Plasma exchange and steroids were discontinued, and eculizumab 900 mg intravenously was initiated.

Prior to eculizumab administration, the patient received meningococcal (MenACWY and MenB), *Haemophilus influenzae* Type b, and pneumococcal vaccinations. As the urgency of his clinical situation did not permit the recommended 2‐week interval between vaccination and eculizumab initiation, prophylactic antibiotics (penicillin V 500 mg orally twice daily) were prescribed for 2 weeks in accordance with published guidelines [28].

## 6. Outcome and Follow‐Up

Following eculizumab initiation, the platelet count normalized within 3 days, rising from a nadir of 9 × 10^9^/L to > 150 × 10^9^/L. LDH declined from 2727 U/L on admission to 637 U/L after plasma exchange and 256 U/L by discharge. Hemodynamics stabilized with successful weaning of norepinephrine, and mentation and overall clinical status improved. Haptoglobin was not rechecked during admission, limiting characterization of the complete hemolytic response.

At discharge, laboratory findings were as follows: white blood cells 11 × 10^9^/L, platelets 225 × 10^9^/L, Hb 7.7 g/dL, mean corpuscular volume 89.6 fL (normal: 80–100 fL), blood urea nitrogen 60 mg/dL, LDH 256 U/L, AST 15 U/L, and ALT 13 U/L. The persistent anemia at discharge reflected both the expected lag in erythropoietic recovery following severe MAHA and the additional contribution of acute gastrointestinal blood loss from the hematemesis and melena at presentation. It is important to acknowledge that the initial Hb decline was not attributable solely to MAHA—the concurrent upper gastrointestinal hemorrhage was a significant contributing factor to both the anemia and the hemodynamic instability on admission. He was discharged on outpatient hemodialysis three times weekly.

He required hemodialysis for a total of 6 weeks, encompassing both his inpatient stay and the outpatient period. Five weeks after discharge, he was transitioned from eculizumab to ravulizumab, a longer‐acting C5, dosed per label for his body weight of 80 kg: a loading dose of 2700 mg intravenously, followed by 3300 mg at 2 weeks, then 3300 mg every 8 weeks thereafter. At that visit, the Invitae panel returned identifying a heterozygous *PLG* variant, c.1735G > A (p.Gly579Arg), classified as a VUS. No clearly pathogenic complement‐gene variant or Factor H autoantibody was identified. Laboratory investigations at this visit also confirmed normalization of complete blood count, complement levels (C3 and C4), and creatinine to near‐baseline, and hemodialysis was formally discontinued.

At two months, the eGFR was approximately 60 mL/min/1.73 m^2^, and 24‐h urine protein was 600 mg. Urinalysis continued to show blood and protein at 6 months. At one year, serum creatinine and eGFR remained normal, although urinalysis showed persistent 3+ hematuria and 24‐h urine protein had decreased to 420 mg. The persistence of hematuria and proteinuria despite preserved renal filtration was consistent with residual glomerular injury and highlights the value of continued urinalysis and quantitative proteinuria monitoring even after recovery of serum creatinine and eGFR.

At one year, he remained clinically well, had returned to full‐time work, and continued ravulizumab with periodic laboratory monitoring. CBC, creatinine, LDH, and C3/C4 remained normal, although hematuria and proteinuria persisted. The long‐term duration of complement inhibition remains under discussion because the *PLG* variant is a VUS and no clearly pathogenic complement variant was identified. The patient elected to continue ravulizumab after discussion of potential relapse risk, treatment burden, infection risk, and cost; monitored discontinuation remains a future option.

## 7. Discussion

The complement system is a critical arm of innate immunity, comprising a network of serum proteins that, upon activation, mediate three principal effector functions: stimulation of inflammatory responses, opsonization of pathogens, and formation of the membrane attack complex (MAC). The alternative pathway is constitutively active at low levels via spontaneous C3 hydrolysis, generating C3b. Under normal circumstances, both fluid‐phase regulators—CFH and CFI—and cell‐surface regulators—CD55, CD59, and MCP—maintain tight homeostatic control, preventing deleterious complement activation on host tissue. When this regulation fails, complement dysregulation leads to endothelial cell damage, microvascular dysfunction, thrombosis, and end‐organ injury, most commonly in the form of renal failure [29]. Cardiac and neurological involvement, while less emphasized, are well‐recognized manifestations of the systemic nature of C‐TMA, and our patient’s Type 2 myocardial infarction at presentation is consistent with this multiorgan phenotype.

Despite harboring a hereditary predisposition from birth, the majority of patients do not develop clinically apparent C‐TMA until adulthood. This reflects the concept of incomplete penetrance: A complement‐activating event such as surgery, pregnancy, or infection may serve as a possible precipitating trigger, unmasking subclinical complement dysregulation and precipitating florid TMA [30, 31]. Importantly, when a triggering condition such as infection is identified, it should not automatically be assumed to be the root cause of TMA—it may instead be a possible precipitant revealing an underlying complement predisposition. In our patient, rotavirus infection may have served as a precipitating trigger, although its role as a direct contributor to the TMA cannot be determined.

Rotavirus is a nonenveloped, double‐stranded RNA virus transmitted via the fecal–oral route and a leading cause of pediatric gastroenteritis worldwide. In developed countries, an attenuated live vaccine is administered in infancy, making symptomatic infection less common in adults. Rotavirus has been associated with hemolytic uremic syndrome, though typically in the context of coinfection with Shiga toxin–producing bacteria. To our knowledge, only one prior case of rotavirus‐induced HUS without coinfection has been reported—a 2‐year‐old child in Turkey [32]. Because this is a single case, the role of rotavirus in the TMA cannot be determined. In this context, genetic testing was pursued to identify a potential underlying predisposition to complement‐mediated disease.

Genetic testing performed using the Invitae Atypical Hemolytic Uremic Syndrome and Thrombotic Microangiopathies Panel (13‐gene panel) identified a heterozygous variant in *PLG*, Exon 14: c.1735G > A (p.Gly579Arg), classified as a VUS (ClinVar Variation ID: 988227). This sequence change substitutes glycine, a neutral and nonpolar residue, with arginine, a basic and polar residue, at Codon 579 of the plasminogen protein—a physicochemically distinct substitution that alters local charge and polarity. The variant is present in population databases at a low frequency (gnomAD 0.04%; rs138728014) and has not been previously reported in individuals with *PLG*‐related conditions. Advanced in silico modeling of protein sequence and biophysical properties performed by Invitae predicted that this missense change is not expected to disrupt *PLG* protein function, with a negative predictive value of 80%. In the absence of functional studies, segregation data, or prior case reports linking this specific variant to TMA, a causal role cannot be asserted.

To understand the biological plausibility of *PLG* variants in TMA more broadly, it is important to note that the *PLG* gene encodes plasminogen, a serine protease zymogen converted to the active enzyme plasmin by tissue plasminogen activator, urokinase, and factor XII. Plasmin plays a central role in fibrinolysis through degradation of fibrin clots, but it also directly cleaves complement components including C1q, C4, and C5, and degrades extracellular matrix proteins including fibronectin, thrombospondin, and von Willebrand factor [33]. Impaired plasmin activity could theoretically disrupt both fibrinolytic homeostasis and complement regulation simultaneously—providing a plausible, if unproven, mechanistic basis for TMA susceptibility at the intersection of the coagulation and complement systems. Whether the specific substitution at Codon 579 (glycine to arginine) affects any of these plasmin functions remains unknown in the absence of functional data.

We nonetheless believe this finding warrants transparent reporting. *PLG* variants have been identified in approximately 4.9% of patients with C‐TMA in a previously reported cohort [34]. The severe renal‐predominant TMA in our patient occurred in a clinical setting compatible with complement‐mediated disease, although the genetic basis cannot be established from the identified VUS. We therefore report the variant with appropriate caution and encourage functional studies and registry‐based data sharing to clarify the significance of *PLG* variants in C‐TMA.

The clinical course of our patient contrasts with the predominant pattern described in the largest published series of *PLG* variant–associated TMA to date, reported by Agarwal et al. in *Research and Practice in Thrombosis and Haemostasis* in 2025 [35]. That single‐institution retrospective series identified six patients with *PLG* pathogenic variants over 12 years, with a median age at first TMA event of 45.5 years, and found that pathogenic *PLG* variant–associated TMA was generally characterized by a poor response to complement blockade therapy. Several patients in that series experienced recurrent TMA episodes despite complement inhibition, and anticoagulation was proposed as an alternative strategy given evidence of coagulation pathway dysregulation in some *PLG*‐associated cases. Our patient, by contrast, achieved a durable response to eculizumab. It is important to note, however, that the variants described in the Cataland series were classified as pathogenic, whereas the variant identified in our patient is a VUS—a distinction that may be clinically meaningful and may partly explain the divergent treatment responses. The reasons for the differing clinical courses cannot be determined from the available data.

Genetic testing and FHAA are important components of the evaluation of C‐TMA, providing diagnostic context, informing relapse risk, and guiding decisions about the duration of complement inhibitor therapy. A critical caveat is that negative genetic testing does not rule out C‐TMA, and results may take several weeks to return depending on the panel and laboratory used. In the acute setting, empiric plasma exchange was initiated while ADAMTS13 testing was pending [3, 28]. It is important to emphasize that this empiric initiation of plasma exchange was appropriate and guideline‐adherent regardless of the subsequent ADAMTS13 result—the therapeutic decision was correct based on the available information at the time. In our patient, ADAMTS13 activity was subsequently found to be above the threshold for severe ADAMTS13 deficiency, making TTP unlikely. The decision to transition to eculizumab was driven primarily by progressive anuric renal failure despite three sessions of plasma exchange, rather than by complement biomarker values alone. It should also be noted that soluble C5b‐9, while elevated in our patient, is a nonspecific marker that cannot establish or exclude C‐TMA in isolation and must be interpreted alongside the full clinical picture.

The subsequent clinical improvement following eculizumab provided additional clinical support for a complement‐mediated mechanism, although the contribution of eculizumab cannot be separated completely from concurrent plasma exchange, corticosteroids, dialysis, and recovery from the systemic illness. Early initiation of terminal complement inhibition with eculizumab has been associated with substantially improved outcomes in C‐TMA. In a genotype‐matched observational cohort, 5‐year ESRD‐free survival was 39.5% in the historical control cohort versus 85.5% among eculizumab‐treated patients with a complement mutation or Factor H autoantibody [36]. In our patient, renal function ultimately recovered after a period of dialysis dependence; however, this single case cannot establish a causal relationship between the timing of eculizumab and the extent of renal recovery.

The case also highlights an important diagnostic challenge that preceded the treatment decision: The initial peripheral smear showed only rare RBC fragments (1 per high‐power field), whereas the repeat smear demonstrated florid fragmentation. The spleen and reticuloendothelial system can clear fragmented RBCs, resulting in deceptively normal or near‐normal early smears in active MAHA [37]. A 2017 retrospective study found delayed schistocyte appearance in one‐third of TTP cases initially misdiagnosed as autoimmune cytopenia [38]. These findings reinforce the importance of serial peripheral blood‐smear review when clinical suspicion for TMA remains high.

This case underscores three practical lessons: TMA may evolve rapidly despite initially limited schistocytosis; empiric plasma exchange is appropriate when TTP is strongly suspected; persistent severe renal disease despite exclusion of TTP and STEC‐HUS should prompt consideration of C‐TMA and potential C5 inhibitor therapy.

## Funding

No funding was received for this manuscript.

## Consent

The patient has provided written informed consent for publication of this case report.

## Conflicts of Interest

The authors declare no conflicts of interest.

## Data Availability

The data that support the findings of this study are available on request from the corresponding author. The data are not publicly available due to privacy or ethical restrictions.
